# The reproducibility of breathing maneuvers as a vasoactive stimulus in the heart: an oxygenation-sensitive resonance imaging study

**DOI:** 10.1186/s12968-023-00983-4

**Published:** 2023-12-27

**Authors:** Elizabeth Hillier, Jason Covone, Matthias G. Friedrich

**Affiliations:** 1 Faculty of Medicine and Health Sciences, McGill University, Montreal, QC Canada; 2 Faculty of Medicine and Dentistry, University of Alberta, Edmonton, AB Canada; 3 Departments of Medicine and Diagnostic Radiology, McGill University, 1001 Decarie Blvd, Montreal, QC H4A 3J1 Canada

**Keywords:** Oxygenation-sensitive cardiovascular magnetic resonance, Reproducibility, Breathing maneuvers

## Abstract

**Background:**

Endothelial dysfunction and impaired oxygenation of the heart is a hallmark of several diseases, including coronary artery disease, hypertension, diabetes, and sleep apnea. Recent studies indicate that oxygenation-sensitive cardiovascular magnetic resonance (OS-CMR) imaging combined with breathing maneuvers may allow for assessing coronary vascular responsiveness as a marker for coronary vascular function in various clinical settings. However, despite the use of OS-CMR in evaluating tissue oxygenation, the reproducibility of these standardized, combined breathing maneuvers as a vasoactive stimulus has yet to be systematically assessed or validated. In this study, we aimed to assess the reproducibility of vasoactive breathing maneuvers to assess vascular function in a population of healthy volunteers.

**Methods:**

Eighteen healthy volunteers were recruited for the study. Inclusion criteria were an age over 18 years and absence of any evidence or knowledge of cardiovascular, neurological, or pulmonary disease. MRI was performed on a clinical 3 T MRI system (MAGNETOM Skyra, Siemens Healthineers, Erlangen, Germany). The OS-CMR acquisition was performed as previously described (1 min hyperventilation followed by a maximal, voluntary breath-hold). Standard statistical tests were performed as appropriate.

**Results:**

Data from 18 healthy subjects was analyzed. The healthy volunteers had a mean age of 42 ± 15 years and a mean BMI of 25.4 ± 2.8 kg/m^2^, with an average heart rate of 72 ± 11 beats per minute, and ten of whom (56%) were female. There were no significant differences between global myocardial oxygenation (%$\Delta$ SI) after hyperventilation (HV1: − 7.82 $\pm$ 5.2; HV2: − 7.89 $\pm$ 6.4, p = 0.9) or breath-hold (BH1: 5.34 $\pm$ 3.1; BH2: 6.0 $\pm$ 3.3, p = 0.5) between the repeated breathing maneuvers. The Bland–Altman analysis showed good agreement (bias: 0.074, SD of bias: 2.93).

**Conclusion:**

We conclude that in healthy individuals, the myocardial oxygenation response to a standardized breathing maneuver with hyperventilation and a voluntary breath-hold is consistent and highly reproducible. These results corroborate previous evidence for breathing-enhanced OS-CMR as a robust test for coronary vascular function.

## Background

Endothelial dysfunction and impaired oxygenation of the heart are hallmarks of several diseases, including coronary artery disease, hypertension, diabetes, and sleep apnea [1, 2]. Over the recent decade, first-pass perfusion cardiac magnetic resonance imaging (CMR) during vasodilator infusion has emerged as a non-invasive, functional assessment of the myocardial vasculature that has good diagnostic and prognostic value in patients presenting with chest pain or otherwise suspected coronary artery disease [3, 4]. It is however hampered by the need for contrast agent injection and a vasodilating agent such as adenosine or regadenoson.

In addition to the inconvenience of intravenous access, side effects such as chest palpitations, headaches, flushing, and anxiety and safety concerns such as induced arrhythmia limit their clinical applicability [5, 6].

As an alternative to such injections or infusions, inhalation of fixed concentrations of carbon dioxide has been previously described. Carbon dioxide inhalation as a vasoactive stimulus takes advantage of the inherent endothelial-dependent vasodilatory properties of carbon dioxide. Although proven to be an effective method, expensive equipment and complex setup limit its application [7–10]. Moreover, the subjectively perceived shortness of breath by induced hypercapnia typically feels acutely threatening.

Specific breathing patterns have recently been used as an effective alternative to injected or inhaled vasodilators to assess coronary vascular responsiveness, specifically in combination with oxygenation-sensitive cardiac magnetic resonance imaging (OS-CMR) [11–13]. Hyperventilation is known to decrease CO_2_ levels, thereby inducing a vasoconstrictive response and a subsequent decrease in relative OS-CMR signal intensity. In contrast, long breath-holds leading to an increase of CO_2_ have been shown to increase myocardial perfusion and thereby myocardial oxygenation that can be quantified by a predictable increase in OS-CMR signal intensity. In coronary vascular dysfunction, this response is blunted [14, 15]. Notably, previous results have demonstrated that preceding the long breath-hold with a period of hyperventilation leads to an increase in myocardial oxygenation that is at least as strong as that induced by intravenous adenosine [11].

Recent studies indicate that OS-CMR imaging in combination with breathing maneuvers therefore may allow for assessing coronary vascular responsiveness as a marker for coronary vascular function in various clinical settings [15, 16]. However, despite the use of OS-CMR in evaluating tissue oxygenation, the reproducibility of these standardized, combined breathing maneuvers as a vasoactive stimulus has yet to be systematically assessed or validated.

In this study, we aimed to assess the reproducibility of vasoactive breathing maneuvers to assess vascular function in a population of healthy volunteers.

## Methods

This study is a prospective, cross-sectional study of healthy controls that received research ethics board approval at the Research Institute of the McGill University Health Centre. The period of participant recruitment was March 2017–August 2019. All subjects provided written informed consent prior to study enrolment.

### Participants

Eighteen healthy volunteers were recruited for the study through public notification. Inclusion criteria were an age over 18 years and absence of any evidence or knowledge of cardiovascular, neurological, or pulmonary disease. Participants were excluded if they had general contraindications to undergo magnetic resonance imaging (MRI), including but not limited to pregnancy, foreign metallic objects, and pacemakers, as well as nicotine consumption within the last six months or unstable hemodynamic conditions.

### Imaging protocol

MR imaging was performed on a clinical 3 T MRI system (MAGNETOM Skyra, Siemens Healthineers, Erlangen, Germany). Participants were asked to refrain from consuming caffeine for 12 h before the exam. Eighteen healthy volunteers successfully completed two repeated breathing maneuvers on CMR using an 18-channel body array coil. OS-CMR images were obtained in a basal and mid-ventricular short-axis slice using a triggered bSSFP sequence (TR/TE = 3.4/1.70 ms, FA = 35, voxel size = 2.0 × 2.0 × 10.0 mm, matrix = 192 × 120, bandwidth = 1302 Hz/Px) as previously described [14].

The OS-CMR imaging protocol involved a baseline OS acquisition, followed by 60 s of hyperventilation paced at 30 breaths/min with a metronome which played into the speaker system of the MRI machine. Immediately following the hyperventilation was a period of voluntary maximal breath-hold, where OS-CMR images were continuously acquired until the participant resumed normal breathing. Participants repeated the breathing maneuver after a period of 180 s of normal breathing to allow the underlying physiology to return to baseline [17]. The study team recorded any adverse effects after the breathing maneuvers.

To ensure adequate performance of the vasoactive breathing maneuver, especially the period of paced hyperventilation, all participants were shown a video demonstrating both proper and improper technique of the hyperventilation prior to entering the MRI scanner. In the scanner, before the breathing maneuver, participants were again reminded of the components of the maneuver. Throughout the performance of the paced hyperventilation, all participants were monitored for compliance through direct visualization of abdominal and thoracic motion through the window of the control room, and by monitoring the rate and amplitude of the respiratory signal from a respiratory belt. If participants were not breathing deep or fast enough, they were provided real-time coaching and guidance from the research team.”.

### Imaging data analysis

All primarily coded images were anonymized to secondary codes before being sent to the readers. All image analysis was performed in a blinded fashion at the McGill University Health Centre.

The OS-CMR images were analyzed using a module for analysing dynamic changes of signal intensity of certified software (cvi42, Circle Cardiovascular imaging, Calgary, Alberta, Canada), with contours drawn manually at end-systole with endocardial and epicardial. Individual segments in an image were entirely excluded if more than 33% of the segment had to be removed due to pre-defined artifacts. Signal intensity changes at the 30 s time point of the breath-hold were analyzed against the first end-systolic OS-image obtained after hyperventilation, as previously described [16].

### Statistical analysis

Continuous variables are reported as mean $\pm$ standard deviation. Categorical variables are reported as frequency distributions. All data sets were assessed for normality with the D’Agostino-Pearson test. A paired Student’s t-test, intra-class and inter-class coefficients of variation (CV) were performed to assess the differences in global myocardial oxygenation values across breathing maneuvers. Correlation between breathing maneuvers was assessed with the Pearson’s correlation coefficient (r) reported. The repeatability of the methodology was assessed using a Bland–Altman analysis. Results were considered statistically significant if the p value was less than 0.05. All statistical analysis was performed using Prism 9 (GraphPad Software, USA).

## Results

Data from 18 healthy subjects was analyzed. The healthy volunteers had a mean age of 42 ± 15 years and a mean BMI of 25.4 ± 2.8 kg/m^2^, with an average heart rate of 72 ± 11 beats per minute, and ten of whom (56%) were female.

All participants completed the protocol. Two participants reported tingling in the fingers after their first period of hyperventilation. No other side effects were reported. All images could be analyzed.

### CMR

#### Reproducibility of myocardial oxygenation

**Fig. 1:**
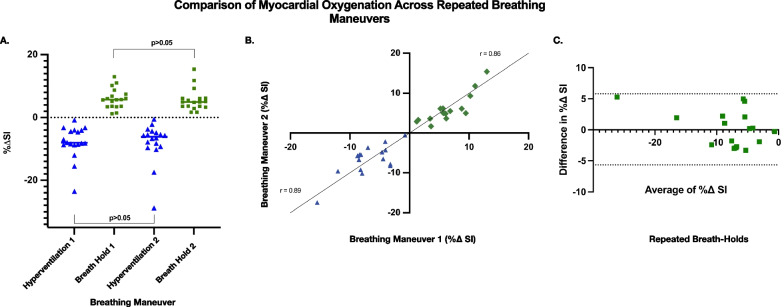
Comparison of myocardial oxygenation after hyperventilation and breath-hold across repeated breathing maneuvers. The percent change in global myocardial signal intensity during hyperventilation is represented in blue, and the breath-hold in green. The latter reflects changes of myocardial blood flow and oxygenation and is known as the Breathing-induced Myocardial Oxygenation Reserve (B-MORE). **A** Percent signal intensity change during hyperventilation and breath-hold for the repeated breathing maneuvers. **B** Correlation graph of repeated measurements of the myocardial oxygenation response to hyperventilation and breath-hold. **C** Bland–Altman plot of repeated measurements of B-MORE

There were no significant differences between global myocardial oxygenation (%$\Delta$ SI) after hyperventilation (HV1: − 7.82 $\pm$ 5.2; HV2: − 7.89 $\pm$ 6.4, p = 0.9) or breath-hold (BH1: 5.34 $\pm$ 3.1; BH2: 6.0 $\pm$ 3.3, p = 0.5) between the repeated breathing maneuvers (Fig. 1A). There was a significant, strong correlation between repeated breathing maneuvers for myocardial oxygenation (%$\Delta$ SI) after both hyperventilation (r = 0.891, p < 0.0001) and breath-hold (r = 0.860, p < 0.0001) between repeated breathing maneuvers (Fig. 1B). The Bland–Altman analysis showed good agreement (bias: 0.074, SD of bias: 2.93) (Fig. 1C).

The mean intra-class CV for global myocardial oxygenation after hyperventilation and breath-hold was 26.2% and 17.9%, respectively. The inter-class CV for global myocardial oxygenation after hyperventilation and breath-hold was 71.9% and 50.3%, respectively.

#### Heart rate

The overall mean heart rate response to hyperventilation ($\Delta \mathrm{HR}$) for all measurements was 15 $\pm$ 7.5 beats per minute. There was no significant $\Delta \mathrm{HR}$ between breathing maneuvers ($\Delta \mathrm{HR}1$: 15 $\pm$ 7.8; $\Delta \mathrm{HR}2$: 16 $\pm$ 7.4, p = 0.7), with a significant correlation in heart rate response between the breathing maneuvers (r = 0.77, p = 0.0002). The mean intra-class CV for $\Delta \mathrm{HR}$ was 18.2% and the inter-class CV was 46.7%.

## Discussion

**Fig. 2:**
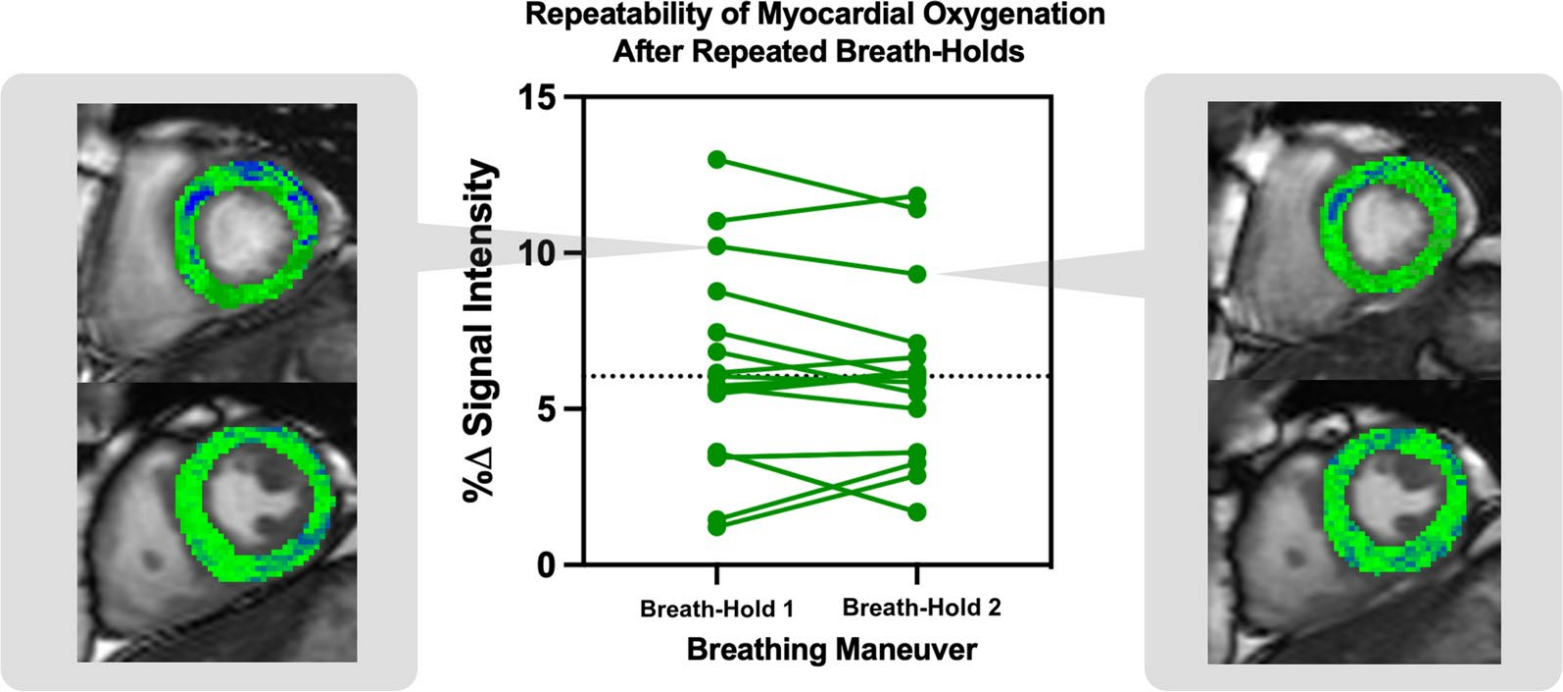
Response of the global breathing-induced myocardial oxygenation reserve to repeated breathing maneuvers in the same healthy volunteer

To our knowledge, this is the first study to assess the reproducibility of a combined breathing maneuver as a vasoactive stimulus in the heart. The present study demonstrated that a combined breathing maneuver is a robust vasoactive stimulus that provides reproducible changes in signal intensity on OS-CMR in healthy individuals (Fig. 2). The reproducibility of myocardial oxygenation reserve was evaluated in 18 healthy volunteers and repeated twice.

The myocardial oxygenation reserve measured in the myocardium during repeated breathing maneuvers was consistent for hyperventilation and breath-hold (p < 0.01). Furthermore, repeated measures of the hyperventilation and breath-hold portions of the breathing maneuver were strongly correlated.

Moreover, there was a reproducible heart rate response to repeated vasoactive stress, with values consistent to those previously published in healthy volunteers [18].

To contextualize our findings, it is important to address the measures of intra- and inter-subject variability to assess the consistency and reliability of the quantitative signal intensity response to vasoactive breathing maneuvers obtained with OS-CMR. The mean and standard deviation of the global myocardial oxygenation response to breath-hold (B-MORE) of our healthy controls are consistent with previously published values [13], suggesting that the variability, both within and between healthy volunteers, observed in our study is in line with previous studies utilizing this technique.

## Limitations

The main limitation of this study is that it does not include any patient participants. Additionally, while our calculated values of intra- and inter-subject variability are relatively high, this may be due to biological variability within participants or OS-CMR signal intensity measurement sensitivity to even subtle physiological changes. The current sample size does not allow us to explore these findings further. Further studies to evaluate the reproducibility of vasoactive breathing maneuvers and OS-CMR in patient populations should be undertaken.

## Conclusions

We conclude that in healthy individuals, the myocardial oxygenation response to a standardized breathing maneuver with hyperventilation and a voluntary breath-hold is consistent and highly reproducible. These results corroborate previous evidence for breathing-enhanced OS-CMR as a robust test for coronary vascular function.

## Data Availability

The dataset analyzed during the current study available from the corresponding author on reasonable request.

## Abbreviations

OS-CMR: Oxygenation-sensitive cardiac magnetic resonance imaging
MRI: Magnetic resonance imaging
CV: Coefficient of variation
HV: Hyperventilation
HR: Heart rate
B-MORE: Breathing induced myocardial oxygenation reserve
